# Transport of solid bodies along tubular membrane tethers

**DOI:** 10.1371/journal.pone.0210259

**Published:** 2019-01-16

**Authors:** D. R. Daniels

**Affiliations:** College of Engineering, Swansea University, Bay Campus, Swansea, United Kingdom; University of California Santa Barbara, UNITED STATES

## Abstract

We study the crucial role of membrane fluctuations in maintaining a narrow gap between a fluid membrane tube and an enclosed solid particle. Solvent flows can occur in this gap, hence giving rise to a finite particle mobility along the tube. While our study has relevance for how cells are able to transport large organelles or other cargo along connecting membrane tubes, known as tunneling nanotubes, our calculations are also framed so that they can be tested by a specific in vitro experiment: A tubular membrane tether can be pulled from a membrane reservoir, such as an aspirated Giant Unilamellar Vesicle (GUV), e.g. using a conjugated bead that binds to the membrane and is held in a laser trap. We compute the subsequent mobility of colloidal particles trapped in the tube, focusing on the case when the particle is large compared to the equilibrium tube radius. We predict that the particle mobility should scale as ∼ *σ*^−2/3^, with *σ* the membrane tension.

## Introduction

There has been a great deal of interest over recent years in the structure and dynamics of fluid membrane tubes. These can be generated by in-vitro tether-pulling experiments [1–7]. It has also been shown that cells exchange enclosed material between themselves via the formation of similar long, narrow fluid membrane tubes known as tunneling nanotubes (TNTs) [8–10]. Such membrane tubes typically possess diameters of 50 nm to 200 nm and can extend over tens of microns. The transport of pathogens between cells using TNTs is also implicated in many important diseases, such as HIV, cancer, bacterial infection, prion, neuronal and immune disorders [8–11]. Bulges are observed in the diameter of TNTs at the position of an enclosed organelle or other object with a size larger than the equilibrium diameter of the enclosing fluid membrane tube [12–15]. Such ‘bulges’ are also observed in the transport of silicon microparticles [16] between cells. Our work may therefore also be relevant to understanding the role of TNTs in disease and drug delivery.

**Fig 1:**
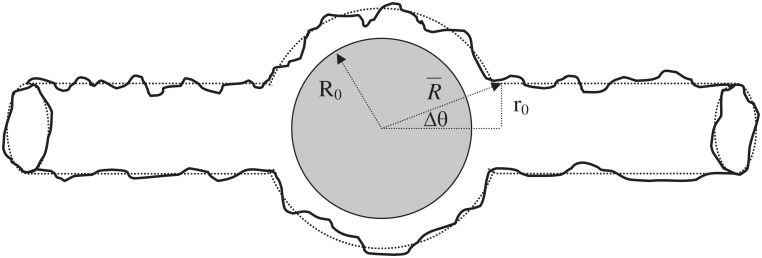
Sketch of a large solid sphere of radius *R*_0_ enclosed in a narrow fluctuating membrane tube. The undeformed section of membrane tube has a radius *r*_0_ ≪ *R*_0_, while the highly deformed portion of membrane enclosing the solid sphere possesses (on average) a radius of $\bar{R}>R_{0}$ due to membrane fluctuations. The degree of wrapping of the solid sphere by the enclosing membrane is characterised by the angle Δ*θ* ≪ 1. (Membrane fluctuation extent shown is exaggerated, for purposes of illustration).

In this work we consider an experimental setup in which a colloidal particle (assumed to be spherical) is trapped inside a tubular tether. This tether is closed at the distal end, near where an elongation force is applied to generate and maintain it, e.g. via a conjugated bead held in a laser trap. Typically the tube is pulled from a Giant Unilamellar Vesicle (GUV) that is aspirated by an attached micropipette, controlling the pressure, and hence the membrane tension. Large tension results in a narrow membrane tube (and vice versa). In-vitro tube diameters are typically in the tens to hundreds of nanometers range and hence can be much narrower than modestly sized colloidal particles. Some of the fluid trapped in the tube between the colloidal sphere and the closed end of the tube must flow past the sphere if it is to move relative to the tube. We consider a tethered tube of fixed length made up of membrane under a surface tension *σ* and with intrinsic rigidity *κ*, see Fig 1. For a large solid sphere enclosed in a narrow fluid membrane tube, the gap in which fluid flows is necessarily small. However, at non-zero temperature an entropic, steric repulsion operates between the membrane and the sphere due to the presence of membrane fluctuations. These fluctuations maintain a finite average gap size between the spherical particle and the enclosing membrane. The existence of such a gap enables fluid to flow around the sphere and therefore endows the sphere with a finite mobility, and corresponding axial diffusion coefficient via the Einstein relation. In this work we estimate the size of this membrane gap self-consistently, and then use this to calculate the drag on a slowly moving enclosed sphere using low Reynolds number hydrodynamics. This allows us to compute the particle mobility. This is found to depend on the membrane tension as ∼ *σ*^−2/3^.

For the interested reader, related works in the literature on the hydrodynamics of membranes, and membrane bound inclusions, can be found in [17–20], for example. Additionally, it is conceivable, within the wider context of cell biology, that the fluid enclosed by the membrane could contain cytoplasm, and hence possibly give a non-Newtonian fluid response [21]. In this work we assume low Reynolds number, Newtonian fluid behaviour, and leave the theoretically challenging possibility of modelling any non-Newtonian fluid response to future work. Moreover, it was recently found in [22] that Newtonian flows were sufficient to describe cytoplasmic streaming in *C. elegans*, for example.

## Results

We calculate the mobility of a spherical particle moving in a membrane tube with an equilibrium radius that is much smaller than that of the moving particle. This corresponds to a membrane tension *σ* that is sufficiently small so that the equilibrium tube radius $r_{0}=\sqrt{\kappa /(2\sigma )}$ is much smaller than the particle radius *R*_0_, with *κ* the membrane rigidity. For typical values of the bending modulus *κ* = 20*k*_*B*_*T* and surface tension *σ* = 10^−5^*Jm*^−2^ (although this can be varied by at least two orders of magnitude by aspiration of the GUV from which the tether is pulled), a typical membrane tube radius is $r_{0}=\sqrt{\kappa /(2\sigma )}∼60\text{nm}$ and therefore even relatively small colloidal particles can fall in the regime of validity of our calculation. Our primary result is that the mobility is reduced in a way that is sensitive to membrane tension. A particle moving in bulk solvent has a diffusion constant that is greater than a similar particle moving in a membrane tube filled with the same solvent by a factor $K=32(\frac{R_{0}^{2}\kappa \sigma }{{\left(k_{{}_{\text{B}}}T\right)}^{2}}{)}^{2/3}$.

## Discussion

We calculate the linear mobility, characterised by the diffusion constant *D*. As usual we assume that all fluid flows are adiabatically slow, such that membrane elasticity and membrane fluctuation contributions completely dominate over any hydrodynamic effects when calculating the membrane gap conformation. This means that the fluctuation force dominates any changes in the hydrostatic pressure at leading order and that we can set *J* and *C* to be constants. We assume that dissipation is dominated by flows inside the tube, as may be confirmed *a posteriori*.

We can also estimate the dissipation due to fluid flow within the membrane as follows. We let the membrane fluid with velocity *u*_*m*_ and viscosity *μ*_*m*_ occupy a region with between $\bar{R}$ and $\bar{R}+h$, where *h* is the membrane thickness. Even though the solvent (water) viscosity *μ* is small (*μ*/*μ*_*m*_ ∼ 10^−3^) the sphere is large compared to the membrane thickness *h*/*R*_0_ ≪ 1 (with *h* ∼ 5*nm*) and this plays a geometrical role, with the dissipation due to flow within the membrane shown to be negligible for the purposes of computing the overall hydrodynamic drag force on the body.

Passive transport of large colloidal bodies bodies enclosed inside membrane tethers is extremely slow, with passive diffusion over distances ∼ 10*μ*m taking ∼ months. This suggests that transport requires active propulsion, such as provided by polymerising fibers of actin or tubulin, molecular motors, or surface tension or pressure differences, leading to membrane or fluid flows, respectively [23]. The work presented here may be relevant to the use of microparticles for novel methods of drug delivery. Most directly, the theoretical work presented here provides testable predictions for the calibration of mobility in tether-pulling experiments. These can be used to directly study the role of membrane fluctuations, something that is otherwise not straightforward to achieve.

For the purposes of the work presented here, we assume from the outset that the particle has already overcome any possibly existing initial entropic barrier effects, and is therefore necessarily well inserted inside the enclosing membrane tube. Naturally, this can be accomplished via direct experimental manipulation, if required, thus obviating the need for any additional entropic cost analyses in this work. Such an interesting and non-trivial entropic cost analysis, suggesting conditions under which a particle might partition within the tether, rather than being excluded from the confinement, is left to future work. Another interesting question is to ask how multiple particles within a single tube might organize to lower the elastic cost, and how any aggregation might affect particle mobilities. Clearly this highly challenging problem is beyond the scope of the work presented here, and is also left to future work. Additionally, how a particle confined in a tube containing phase separating membrane molecules, which produce domains of differing bending rigidities and thus different tensions, might repartition to minimize the energy, is another interesting question one might like to address. Again, this highly non-trivial problem is beyond the scope of the work presented here, and is thus similarly left to future work.

## Model

### Self-consistent calculation of the membrane gap

In the following section we outline a self consistent mean field analysis of the radial membrane fluctuations. The purpose of this is to calculate the average size of the gap that exists between the spherical particle and the fluctuating membrane; in some places the gap will be smaller and others it will naturally be larger. Our approach will be to use the average gap size as an approximation for a (constant) gap size, everywhere around the sphere. In our model the average radial extent of the membrane is governed by membrane fluctuations that drive it slightly away from the surface of the enclosed spherical particle. A similar approach has proved highly successful for planar membranes [24, 25], as well as membrane tubes [26].

The membrane fluctuates about a spherical shape around the particle (bulge) except near the two “necks” where it joins smoothly onto the walls of the membrane tube, see Fig 1. In order to describe the energetics of the membrane we write the Hamiltonian *H* = *H*_*E*_ + *H*_*S*_, with

$$\begin{array}{c} H_{E}=\int [\sigma +\frac{\kappa }{2}c^{2}]\;\sqrt{g}\;d\phi d\theta \end{array}$$ (1)

and

$$\begin{array}{c} H_{S}=\int [A+J{\bar{R}}^{2}(R\left(\phi ,\theta \right)-\bar{R})+\frac{C}{2}(R\left(\phi ,\theta \right)-\bar{R}{)}^{2}]d\phi \;\text{sin}\;\theta d\theta \end{array}$$ (2)

Here *H*_*E*_ is the usual Hamiltonian for membrane elasticity [25, 27], containing both surface tension (*σ*) and rigidity (*κ*) controlled terms. The mean curvature is given by *c*, and *g* is the determinant of the metric tensor det(*g*_*ab*_). The steric part, *H*_*S*_, contains a harmonic potential with strength C that confines the size of the membrane fluctuations in a narrow region around the average membrane sphere radius $\bar{R}$. It also contains a fluctuation pressure with strength *J*, which controls the average radius $\bar{R}$ of the membrane sphere. This term can also be used to include any hydrostatic or osmotic pressure differences between the inside and outside of the membrane, although we set these to zero for clarity in what follows. Additionally, *H*_*S*_ contains a term involving *A* which is convenient for normalisation of the steric potential. This, most general harmonic potential, will be used to model the steric interactions between our membrane and enclosed spherical particle by way of a mean-field approach. As we are dealing in this work with an almost completely enveloped spherical particle, we can approximate the energy by computing the membrane Hamiltonian over the entire sphere. An analogous treatment has proven to be remarkably successful in describing the steric repulsion between flat membranes [24, 25], as well as between membrane tubes and an enclosed rod [26]. We proceed from Eq (1) by writing $R\left(\phi ,\theta \right)=\bar{R}+\delta R\left(\phi ,\theta \right)$ and expanding the energy *H* to quadratic order [28] in the radial perturbation *δR*(*ϕ*, *θ*) about the average membrane radius $\bar{R}$. Using spherical harmonics, we write $\delta R\left(\phi ,\theta \right)=\sum_{lm}\delta R_{lm}Y_{l}^{m}\left(\phi ,\theta \right)$, which yields the total membrane energy as a perturbative expansion *H* = *H*_0_ + *δH* + *δ*^2^*H* + … with

$$\begin{array}{ccc} H_{0} & = & 8\pi \kappa +4\pi \sigma {\bar{R}}^{2}+4\pi A\\ \delta H & = & 0\Rightarrow \left\langle \delta R\right\rangle \;=0\Rightarrow J=-2\sigma /\bar{R}\\ \delta^{2}H & = & \frac{1}{2}\sum_{lm}{\left|\delta R_{lm}\right|}^{2}K_{lm} \end{array}$$ (3)

involving a kernel

$$\begin{array}{c} K_{lm}=\left(l-1\right)\left(l+2\right)(\frac{\kappa }{{\bar{R}}^{2}}l\left(l+1\right)+\sigma )+C \end{array}$$ (4)

The first order perturbative contribution is required to vanish so that $\bar{R}$ indeed represents the true average (or ground state) membrane radius [28]. This condition then implies (from Eq (3)) that the fluctuation pressure is $J=-2\sigma /\bar{R}$, as required to satisfy Laplace’s law. The quadratic fluctuations in the radial displacement (around $\bar{R}$), contribute at order *δ*^2^*H*, and depend on the strength of the harmonic potential in Eq (1), via the parameter C which is established as follows. The presence of the enclosed solid spherical particle sterically constrains the membrane radius, *R*(*ϕ*, *θ*), to remain always greater than the particle radius *R*_0_, see Fig 1. The mean squared amplitude of the fluctuations, 〈*δR*^2^〉, depends on the parameter C, as can be seen from Eq (3), which we must determine self consistently. In employing a harmonic potential, controlled by the parameter C, we adopt an approximate phenomenological treatment of the steric interactions.

By integrating out the membrane fluctuations, the free energy of the tube *F* = *H*_0_ + Δ*F* can be shown to involve the correction term:

$$\begin{array}{c} \Delta F=\frac{1}{2}k_{{}_{\text{B}}}T\sum_{lm}\text{log}(K_{lm}) \end{array}$$ (5)

We can now physically motivate an explicit choice for the parameter *A* as follows. We aim to calculate the free energy difference between the case when the enclosed spherical particle is present and when it is absent (and the membrane is unconstrained). In the latter case the steric harmonic potential (of strength C) vanishes, as do terms involving C that appear in *K*_*nm*_. Thus we choose the parameter *A* so that in the limit C→0 we obtain Δ*F* → 0 for consistency. We must then choose

$$\begin{array}{c} A=-\frac{1}{8\pi }k_{{}_{\text{B}}}T\sum_{lm}\text{log}(K_{lm|C=0}) \end{array}$$ (6)

After we have integrated out all radial membrane fluctuations we therefore obtain

$$\begin{array}{c} F=8\pi \kappa +4\pi \sigma {\bar{R}}^{2}+\frac{1}{2}k_{{}_{\text{B}}}T\sum_{lm}\text{log}(\frac{K_{lm}}{K_{lm|C=0}}) \end{array}$$ (7)

We can now state quantitatively the physical condition that we wish to impose on our membrane to mimic the steric influence of the enclosed solid sphere (with radius *R*_0_):

$$\begin{array}{c} \bar{R}-\sqrt{\left\langle \delta R^{2}\right\rangle }=R_{0} \end{array}$$ (8)

This gives the necessary self-consistency condition for the strength of the harmonic potential given that

$$\begin{array}{c} \left\langle {\delta R}^{2}\right\rangle =\frac{1}{4\pi }k_{{}_{\text{B}}}T\sum_{lm}K_{lm}^{-1} \end{array}$$ (9)

In order to calculate the average radius $\bar{R}$ for the membrane tube we merely need to minimise *F* by setting $\frac{\partial F}{\partial \bar{R}}=0$.

For a large sphere inside a narrow tube, the average radius of the membrane is almost equal to the radius of the enclosed spherical particle ($\bar{R}/R_{0}≃1$). The steric effects of the sphere in this limit should therefore be very strong, and the strength of the self-consistent, confining, harmonic potential becomes very large ($C{\bar{R}}^{2}/\kappa \gg 1$). Using $\sum_{m=-l}^{m=l}=2l+1$, we can approximate the resultant sum over *l* in Eq (9) as an integral by defining a new variable *ρ* = *l*^2^, such that:

$$\begin{array}{ccc} \frac{{\left(\bar{R}-R_{0}\right)}^{2}}{R_{0}^{2}} & ≃ & \frac{1}{4\pi \kappa }k_{{}_{\text{B}}}T\int_{0}^{\infty }d\rho \frac{1}{\rho^{2}+\frac{C{\bar{R}}^{2}}{\kappa }}\\ & \to & \frac{1}{8}k_{{}_{\text{B}}}T\sqrt{\frac{1}{\kappa C{\bar{R}}^{2}}}\;\text{as}\;C\to \infty \end{array}$$ (10)

Hence in this narrow gap limit, $C=\frac{R_{0}^{2}}{64\kappa }\frac{{\left(k_{{}_{\text{B}}}T\right)}^{2}}{{\left(\bar{R}-R_{0}\right)}^{4}}$, to leading order. Substituting this value of C into Eq (7), and approximating the sum required by an integral as in Eq (10), we obtain the following result to leading order

$$\begin{array}{c} F=8\pi \kappa +4\pi \sigma R_{0}^{2}+8\pi \sigma R_{0}\left(\bar{R}-R_{0}\right)+{\left(k_{{}_{\text{B}}}T\right)}^{2}\frac{\pi R_{0}^{2}}{16\kappa }\frac{1}{{\left(\bar{R}-R_{0}\right)}^{2}} \end{array}$$ (11)

A contribution to the energy that scales as the inverse squared distance, similar to the one appearing here, is well known for planar membranes at small inter-membrane separation [24, 29–31], as well as membrane tubes in close proximity to an enclosed rod [26].

Minimising Eq (11) w.r.t. $\bar{R}$, we find to leading order:

$$\begin{array}{c} \bar{R}=R_{0}+(\frac{R_{0}{\left(k_{{}_{\text{B}}}T\right)}^{2}}{64\kappa \sigma }{)}^{\frac{1}{3}} \end{array}$$ (12)

In closing, note that the distal portions of membrane tube, unaffected by the presence of the enclosed large spherical particle, can easily be shown [26] to possess a cylindrical radius of $r_{0}=\sqrt{\frac{\kappa }{2\sigma }}$.

### Fluid hydrodynamics

Here we analyse the fluid hydrodynamics around the moving particle within the tube. This will allow us to calculate the diffusion constant of the spherical particle. The low Reynolds number hydrodynamics of the fluid within the membrane tube is governed by Stokes’ equation, along with the constraint of incompressibility [32–35]:

$$\begin{array}{c} -\nabla p+\mu \nabla^{2}u=0\\ \nabla \cdot u=0 \end{array}$$ (13)

with fluid velocity u, hydrostatic pressure *p*, and viscosity *μ*. Utilising symmetry considerations, we work in spherical polar coordinates, and assume that *u*_*ϕ*_ = 0, and *∂***u**/*∂ϕ* = 0. In the ‘narrow-gap’ approximation of interest here, we can also assume that *u*_*R*_ = 0, such that the fluid flow in the gap can be described by the component *u*_*θ*_ alone. Utilising this approximation, and taking into account the geometrical setup of our problem, Eq (13) becomes:

$$\begin{array}{c} -\frac{1}{R}\frac{\partial p}{\partial \theta }+\frac{\mu }{R^{2}}\frac{\partial }{\partial R}(R^{2}\frac{\partial u_{\theta }}{\partial R})=0\\ \frac{1}{R\;\text{sin}\;\theta }\frac{\partial }{\partial \theta }(u_{\theta }\;\text{sin}\;\theta )=0 \end{array}$$ (14)

The incompressibility condition in Eq (14) can be satisfied straightforwardly by defining $u_{\theta }=\frac{\Phi (R)}{\text{sin}\;\theta }$, such that the *z* component of the flow (along the tube axis) becomes simply: *u*_*z*_ = −*u*_*θ*_ sin *θ* = −Φ(*R*). Using this expression for *u*_*θ*_, we find that the equation governing the fluid flow and pressure can now be re-arranged into the following form:

$$\begin{array}{c} \text{sin}\;\theta \frac{\partial p}{\partial \theta }=\frac{\mu }{R}\frac{\partial }{\partial R}(R^{2}\frac{\partial \Phi }{\partial R})=a \end{array}$$ (15)

The most general solution to Eq (15) can easily be shown to be: $\Phi \left(R\right)=\frac{a}{2\mu }R-\frac{c}{R}+d$, and $p\left(\theta \right)=b+\frac{a}{2}\;\text{ln}\;(\frac{1-\text{cos}\;\theta }{1+\text{cos}\;\theta })$, where *a*, *b*, *c*, and *d* are constants. For convenience, we choose to work in the lab frame with respect to which the enclosed solid sphere is moving. Furthermore, we need to take into account the fluid flow in the spherical gap between the enclosed sphere and the membrane, and the membrane fluid flow in the spherically deformed membrane, the *z* component of which is written as *u*_*m*_, such that the boundary conditions satisfied by the fluid are as follows:

$$\begin{array}{ccc} u_{z}{|}_{R=R_{0}} & = & u_{0}\\ u_{z}{|}_{R=\bar{R}} & = & u_{m} \end{array}$$ (16)

The membrane tube is held stationary at its end by, e.g. a conjugated bead held in an optical trap. This means that the membrane tube is not growing in length, and that the sum of the length of tube in front of the moving sphere and the length behind it must always add up to a constant, the total tube length. The sphere is assumed to be moving such that the tube length in front of the moving sphere decreases while the tube length behind it increases. The tube and its contents remain stationary everywhere except very close to the sphere, while the enclosed fluid and membrane must flow around the sphere. If the length of tube in front of the sphere is *L*_0_ at time *t* = 0 then it is *L*(*t*) = *L*_0_ − *u*_0_*t* at some later time *t*. Thus the volume in the leading tube is $\pi r_{0}^{2}L\left(t\right)$ and depends on time, which demands a flow around the sphere to balance volume (with a similar argument for the membrane flow).

The surface tension gradient, expressed as a difference between the tension in the leading and trailing tubes, is *O*(*u*_0_), i.e. small. Because the membrane flow is effectively one dimensional, the membrane velocity is entirely determined by (i) membrane incompressibility (and geometry) and (ii) the radii of the leading and trailing tubes, from which the membrane is extracted/deposited so as to leave the membrane in both tubes stationary everywhere (except around the sphere). This means that the membrane flow can only depend on the surface tension difference via the leading and trailing tube radii, and here these are both of order *r*_0_(1 + *O*(*u*_0_)), i.e. equal to *r*_0_ to leading order. Thus we can safely ignore membrane tension gradients set up by the flow itself, and therefore any concomitant differences in the leading or trailing tube radii.

In the lab frame, the fluid is stationary everywhere in both leading and trailing tubes. However, due to the change in membrane tube length in front of the moving sphere this means that there must be a volumetric back-flow of fluid in the gap around the sphere ($=u_{0}\pi r_{0}^{2}$), in order to avoid fluid accumulating in the leading tube. Thus, volumetric flow balance leads to:

$$\begin{array}{c} \pi r_{0}^{2}u_{0}=2\pi \int_{R_{0}}^{\bar{R}}u_{z}\left(R\right)RdR \end{array}$$ (17)

The important point to note in Eq (17) is that from the definition of the volumetric flow rate being: $\int_{0}^{2\pi }d\phi \int_{R_{0}}^{\bar{R}}u_{\theta }\;\text{sin}\;\theta RdR$, the integrand in Eq (17) is independent of the angle *θ*.

Additionally, in the lab frame, the membrane fluid is stationary everywhere along both leading and trailing tubes. However, this means that there must also be a volumetric back-flow of membrane around the sphere (= *u*_0_2*πr*_0_*h*), with *h* the membrane thickness, to avoid membrane accumulating on the leading tube. Volumetric flow balance in this case therefore leads to:

$$\begin{array}{c} 2\pi u_{0}r_{0}h=2\pi \int_{\bar{R}}^{\bar{R}+h}u_{z}\left(R\right)RdR \end{array}$$ (18)

which we evaluate in the limit where the membrane thickness *h* becomes vanishingly small.

By utilising all of the above boundary conditions, we can find all the integration constants required in order to compute the drag force on the membrane enclosed sphere:

$$\begin{array}{ccc} \frac{a}{2\mu } & = & \frac{3u_{0}}{{\left(\bar{R}-R_{0}\right)}^{3}}(r_{0}^{2}-\left(\bar{R}-R_{0}\right)\left(R_{0}+r_{0}\right))\\ b & = & \frac{1}{2}(p(\Delta \theta )+p(\pi -\Delta \theta ))\\ c & = & -\frac{R_{0}u_{0}}{{\left(\bar{R}-R_{0}\right)}^{3}}(3r_{0}^{2}\bar{R}-\left(\bar{R}-R_{0}\right)(r_{0}\left(2\bar{R}+R_{0}\right)+\bar{R}\left(\bar{R}+2R_{0}\right)))\\ d & = & -\frac{u_{0}}{{\left(\bar{R}-R_{0}\right)}^{3}}(3r_{0}^{2}\left(\bar{R}+R_{0}\right)-2\left(\bar{R}-R_{0}\right)(r_{0}\left(\bar{R}+2R_{0}\right)+R_{0}\left(2\bar{R}+R_{0}\right))) \end{array}$$ (19)

where Δ*θ* ≈ *r*_0_/*R*_0_ is the small angle subtended by the neck of the tube, see Fig 1. In terms of these constants, the fluid velocity and pressure become:

$$\begin{array}{ccc} u_{\theta }\left(R\right) & = & \frac{1}{\text{sin}\;\theta }\frac{u_{0}}{{\left(\bar{R}-R_{0}\right)}^{3}}(3R(r_{0}^{2}-\left(\bar{R}-R_{0}\right)\left(R_{0}+r_{0}\right))\\ & + & \frac{R_{0}}{R}(3r_{0}^{2}\bar{R}-\left(\bar{R}-R_{0}\right)(r_{0}\left(2\bar{R}+R_{0}\right)+\bar{R}\left(\bar{R}+2R_{0}\right)))\\ & - & \left(3r_{0}^{2}\left(\bar{R}+R_{0}\right)-2\left(\bar{R}-R_{0}\right)(r_{0}\left(\bar{R}+2R_{0}\right)+R_{0}\left(2\bar{R}+R_{0}\right)))\right)\\ p(\theta ) & = & \frac{1}{2}(p(\Delta \theta )+p(\pi -\Delta \theta ))+\frac{3u_{0}\mu }{{\left(\bar{R}-R_{0}\right)}^{3}}(r_{0}^{2}-\left(\bar{R}-R_{0}\right)\left(R_{0}+r_{0}\right))\;\text{ln}\;(\frac{1-\text{cos}\;\theta }{1+\text{cos}\;\theta }) \end{array}$$ (20)

### Calculation of drag

The drag force *f*_0_ is determined by the integration of the fluid stress components (*T*_*RR*_, *T*_*Rθ*_) over the surface of the solid sphere (*R* = *R*_0_) as:

$$\begin{array}{c} f_{0}=2\pi R_{0}^{2}\int_{\Delta \theta }^{\pi -\Delta \theta }d\theta \;\text{sin}\;\theta (T_{RR}\;\text{cos}\;\theta -T_{R\theta }\;\text{sin}\;\theta ) \end{array}$$ (21)

where *T*_*RR*_ = −*p*(*θ*), as given in Eq (20), and $T_{R\theta }=\mu R\frac{\partial }{\partial R}\left(u_{\theta }/R\right)$ is given as:

$$\begin{array}{ccc} T_{R\theta } & = & \frac{\mu }{\text{sin}\;\theta }\frac{u_{0}}{{\left(\bar{R}-R_{0}\right)}^{3}}(-\frac{2R_{0}}{R^{2}}(3r_{0}^{2}\bar{R}-\left(\bar{R}-R_{0}\right)(r_{0}\left(2\bar{R}+R_{0}\right)+\bar{R}\left(\bar{R}+2R_{0}\right)))\\ & + & \frac{1}{R}(3r_{0}^{2}\left(\bar{R}+R_{0}\right)-2\left(\bar{R}-R_{0}\right)(r_{0}\left(\bar{R}+2R_{0}\right)+R_{0}\left(2\bar{R}+R_{0}\right)))) \end{array}$$ (22)

Evaluating the integrals required in Eq (21), we find to leading order, assuming $r_{0}^{2}/R_{0}^{2}<\left(\bar{R}-R_{0}\right)/R_{0}$:

$$\begin{array}{c} f_{0}=-6\pi \mu u_{0}R_{0}\frac{2R_{0}^{2}}{{\left(\bar{R}-R_{0}\right)}^{2}}(\text{cos}\left(\Delta \theta \right)+\frac{1}{2}{\text{sin}}^{2}\left(\Delta \theta \right)\;\text{ln}\;(\frac{1-\text{cos}\;\Delta \theta }{1+\text{cos}\;\Delta \theta })) \end{array}$$ (23)

where sin Δ*θ* = *r*_0_/*R*_0_, and $r_{0}=\sqrt{\frac{\kappa }{2\sigma }}$. Using the relationship *f*_0_ = −*ξu*_0_, and inserting in Eq (23): $\bar{R}-R_{0}=(\frac{R_{0}{\left(k_{{}_{\text{B}}}T\right)}^{2}}{64\kappa \sigma }{)}^{\frac{1}{3}}$, from Eq (12), we can write for the drag ratio *K* = *ξ*/*ξ*_0_:

$$\begin{array}{c} K=\xi /\xi_{0}=32(\frac{\kappa R_{0}^{2}\sigma }{{\left(k_{{}_{\text{B}}}T\right)}^{2}}{)}^{2/3}(\text{cos}\left(\Delta \theta \right)+\frac{1}{2}{\text{sin}}^{2}\left(\Delta \theta \right)\;\text{ln}\;(\frac{1-\text{cos}\;\Delta \theta }{1+\text{cos}\;\Delta \theta })) \end{array}$$ (24)

where the free, geometrically unhindered, 3d bulk friction constant is given as usual by: *ξ*_0_ = 6*πμR*_0_ [34]. To leading order in Δ*θ* ≪ 1, corresponding to particles larger than the tube radius, this simplifies dramatically to:

$$\begin{array}{c} K=\xi /\xi_{0}=32(\frac{\kappa R_{0}^{2}\sigma }{{\left(k_{{}_{\text{B}}}T\right)}^{2}}{)}^{2/3} \end{array}$$ (25)

Note that in Eq (25) the drag ratio depends on the membrane tension as ∼ *σ*^2/3^. This result for the drag provides an experimentally testable prediction. Furthermore, the diffusion constant *D* is given by *D* = *k*_*B*_*T*/*ξ* = *D*_0_/*K*, where *D*_0_ = *k*_*B*_*T*/6*πμR*_0_ [34], which leads to a diffusion constant correspondingly (much) smaller than that for free 3d bulk diffusion in the same fluid. Additionally, the algebraic expression Eq (25), explicitly demonstrates the dependence of mobility on viscosity, such that the effect of higher viscosities than pure water, because of the inclusion of cytoskeleton and cytoplasmic soluble macromolecules (e.g. crowding agents), can easily be taken into account.

For typical fluid membranes we have *κ* ∼ 20*k*_*B*_*T*, and we can investigate how our main result (given by Eq (25)) behaves in the most physiologically relevant particular regimes as follows. Given that most, generic, biophysical cargo (as depicted in Fig 1) possess a range of sizes from *R*_0_ ∼ 0.1 − 10*μm* [11], while most biomembranes have surface tensions between *σ* ∼ 10^−5^ − 10^−4^*Jm*^−2^, we find that our drag ratio lies in the range *K* ∼ 2 × 10^3^ − 4 × 10^6^. The above, experimentally accessible, range of values therefore encompass a host of individual case studies, or particular situations, such as (in vivo [8–16]) the transport of mitochondria (∼ 0.5 − 1*μm*), small organelles (∼ 0.1*μm*), pathogens (∼ 0.1*μm*), viruses (∼ 0.1*μm*), silicon microparticles (∼ 1*μm*), or other large cargo (∼ 1*μm*) in tunneling nanotubes (TNTs), as well as (in vitro) the transport of large colloidal particles or microbeads (∼ 10*μm*) in membrane tether-pulling experiments [1–7], for example. In addition, via Eq (12), we can calculate using the typical parameters quoted above the expected average size of the gap that exists between the spherical particle and the fluctuating membrane to be ∼1 − 10*nm*. This calculated gap size compares favourably with the analogous value of ∼ 3*nm* obtained from experimental work on supported lipid bilayers [36].
